# Characterization of the Serine Protease TlSP1 from *Trichoderma longibrachiatum* T6 and Its Function in the Control of *Heterodera avenae* in Wheat

**DOI:** 10.3390/jof10080569

**Published:** 2024-08-12

**Authors:** Xiujuan Wang, Shuwu Zhang, Bingliang Xu

**Affiliations:** 1 College of Plant Protection, Gansu Agricultural University, Lanzhou 730070, China; 13893386785@163.com; 2 Gansu Provincial Biocontrol Engineering Laboratory of Crop Diseases and Pests, Lanzhou 730070, China; 3 Gansu Provincial Key Laboratory of Arid Land Crop Science, Gansu Agricultural University, Lanzhou 730070, China

**Keywords:** *Trichoderma longibrachiatum* T6, *Heterodera avenae*, serine protease, heterologous, expression, induced resistance

## Abstract

Serine protease is an extracellular protease secreted by biocontrol fungi that can effectively control nematode diseases by degrading nematode eggshells and enhancing plant resistance. *Trichoderma longibrachiatum* T6, an important biocontrol fungus, has been demonstrated to effectively parasitize and degrade *Heterodera avenae* cysts, eggs, and second-stage juveniles (J2s). However, the genes that encoding serine protease and their functions in *T. longibrachiatum* T6 have not been thoroughly investigated. In this study, we successfully cloned and sequenced the serine protease gene *TlSP1* in *T. longibrachiatum* T6. Our results revealed that the expression level of the *TlSP1* gene was induced and significantly increased in *T. longibrachiatum* T6 after inoculation with *H. avenae* cysts. The full-length sequence of the coding region (CDS) of *TlSP1* gene was 1230 bp and encoded a protein consisting of 409 amino acids. Upon the transformation of the *TlSP1* gene into *Pichia pastoris* X33, the purified recombinant TlSP1 protein exhibited optimal activity at a temperature of 50 °C and pH 8.0. Following 4–10-day of treatment with the purified recombinant TlSP1 protein, the eggshells and content were dissolved and exuded. The number of nematodes invading wheat roots was reduced by 38.43% in the group treated with both TlSP1 and eggs on one side (P1+N) compared to the control group, while the number of nematodes invading wheat roots was reduced by 30.4% in the TlSP1 and eggs two-sided treatment group (P1/N). Furthermore, both the P1+N and P1/N treatments significantly upregulated genes associated with defense enzymes (*TaPAL*, *TaCAT*, *TaSOD*, and *TaPOD*), genes involved in the lignin synthesis pathway (*TaC4H*, *Ta4CL2*, *TaCAD1*, and *TaCAD12*), and salicylic acid (SA)-responsive genes (*TaNPR1*, *TaPR1*, and *TaPR2*) and led to the high expression of jasmonic acid (JA)-responsive genes (*TaPR4*, *TaOPR3*, and *TaAOS2*). This study has highlighted the significant role of the *TlSP1* gene in facilitating *H. avenae* eggshells’ dissolution, preventing nematode invasion in the host plant, and boosting plant resistance in wheat.

## 1. Introduction

Plant parasitic nematodes (PPNs) represent a major threat to the agricultural production of different crops worldwide [1]. However, the most important nematodes in terms of crop losses are sedentary endoparasites, the root-knot (*Meloidogyne* spp.) and cyst nematodes (*Heterodera* and *Globodera* spp.) [2]. The economic losses due to their damage to global agricultural production are estimated to be as high as USD 157 billion per year [3]. Historically, chemical nematicides have served as the primary interventions against plant nematode infestations. However, extensive chemical application has fostered nematode resistance and contributed to severe environmental contamination, posing risks to human health. Consequently, many highly toxic nematicides have faced prohibition or usage restrictions [4]. Biological control has gradually become one of the safest and most effective and important measures for the prevention and treatment of plant parasitic nematode diseases, as it can effectively reduce the use of chemical pesticides and mitigate environmental pollution. Filamentous fungi, particularly *Trichoderma* spp., mycorrhizal fungi, and endophytic fungi, represent a promising biocontrol alternative [5,6]. For instance, the secondary metabolites produced by *T. asperelloides* and *T. pseudoharzianum* have shown effectiveness in inhibiting the hatching of *M. incognita* eggs and causing the death of second-stage juveniles (J2s) [7]. Moreover, spores, mycelia, and metabolites from *T. harzianum* and *T. asperelloides* have demonstrated strong inhibitory effects on *M. javanica* in *Solanum lycopersicum* [8]. Subsequent research has revealed that *T. harzianum* can trigger resistance against root-knot nematodes by enhancing the synthesis of secondary metabolites, defense-related enzyme activity, and the salicylic acid (SA) and jasmonic acid (JA) pathways in *S. lycopersicum* [9]. In our previous study, *T. longibrachiatum* T6 exhibited strong parasitic and lethal effects on the J2s and eggs of *H. avenae* in wheat [10,11]. However, there is a limited amount of research on the molecular mechanisms of *Trichoderma* in controlling *H. avenae* in wheat.

Serine proteases are significant extracellular enzymes, known for their biocontrol effectiveness in degrading fungal cell walls, nematode eggshells, and insect cuticles [12,13]. They have demonstrated efficacy against the pathogen of citrus black spot (*Guignardia citricarpa*) [14] and in neutralizing pest like the yellow mealworm (*Tenebrio molitor*) [13]. The interaction between *Trichoderma* and nematodes stimulates protease production, thereby enhancing parasitic and lethality [3,15,16]. Recent advancements have highlighted the potential of serine proteases in reducing nematode populations; for instance, PRA1 from *T. harzianum* [17] and SprT from *T. pseudokoningii* [18] specifically target nematode eggs. Furthermore, the *SL41* gene from *Aspergillus hartzii* and SP1 from *Bacillus licheniformis* W10 have been shown to confer plant resistance against pathogens and modulate disease defense pathways [19]. Additionally, the inhibitory and biocidal functions of the serine proteases ThPRB1 from *T. harzianum* and TvSP1 from *T. virens* have been investigated [20,21]. The SP1 serine protease, derived from *Bacillus licheniformis* W10, impedes *Phomopsis amygdali* and enhances tobacco’s disease resistance through the SA and JA pathways [22]. Our previous study indicated that the serine protease activity was upregulated during the parasitization of *H. avenae* cysts by *T. longibrachiatum* T6. However, investigations into the key serine protease genes of *T. longibrachiatum* T6 that degrade *H. avenae* eggshells, inhibit hatching, and induce resistance have not yet been reported. In particular, the characterization, bioactivity, and function of the serine proteases produced by *T. longibrachiatum* T6 remain unclear.

To address this gap in knowledge, the current study focused on cloning the *TlSP1* gene from *T. longibrachiatum* T6 and characterizing the properties of the recombinant TlSP1 protein after expressing it heterologously in *Pichia pastoris*. Additionally, this study assessed the impact of TlSP1 on *H. avenae* eggs and analyzed the expression of defense-related genes in wheat following TlSP1 application. The goal was to analyze the mechanism of TlSP1-induced wheat resistance to cyst nematodes and provide a theoretical foundation for developing the TlSP1 protein and the formulation of environmentally friendly plant disease prevention and control strategies.

## 2. Materials and Methods

### 2.1. Fungus Inoculum and H. avenae Preparation

The *T. longibrachiatum* T6 strain (CGMCC No. 13183) was obtained from the Laboratory of Plant Virology and Molecular Biology, Gansu Agricultural University, and incubated for 7 days on potato dextrose agar (PDA) medium. Soil from the rhizosphere of wheat with a high incidence of *H. avenae* was collected from Anhui Province, China. pPICZαA and *Pichia pastoris* X33 were purchased from Invitrogen (Thermo Fisher Scientific, Waltham, MA, USA).

### 2.2. Cloning and Sequence Analysis of TlSP1 Gene from T. longibrachiatum T6

The *T. longibrachiatum* T6 mycelia parasitized on the surfaces of the cysts were collected at 72 h after inoculation. All samples were immediately frozen in liquid nitrogen and stored at −80 °C for backup. Total RNA extraction was performed using the E.Z.N.A. Fungal RNA Kit (OMEGA Bio-Tek, Norcross, GA, USA), followed by cDNA synthesis with the RevertAid First Strand cDNA Synthesis Kit (Invitrogen). Specific primers were designed based on the sequenced *T. longibrachiatum* serine protease gene in GenBank (accession number: KZ679129.1) (*TlSP1-*F: ATGTACATGGCCAGCCTCCGT and *TlSP1-*R: AGCACTGTTTCCGTTGAAG) for the cloning of the coding sequence (CDS) of the gene. The PCR products were analyzed by agarose gel electrophoresis, purified using the Agarose Gel DNA Extraction Kit (Takara, Dalian, China), cloned into the pMD19-T vector (Takara), and sequenced by Sangon Biotech., Shanghai, China. The physicochemical properties, transmembrane structural domains, signal peptides, and secondary structures were analyzed using bioinformatics methods, as previously described [23]. The homologous sequence of *TlSP1* was obtained from the NCBI database, and the phylogenetic tree was constructed using the MEGA 11.0 software with the neighbor-joining (NJ) method and a bootstrap value of 1000. The sequences and GenBank accession numbers for all proteins are as follows: LpVER112 (Q68GV9.1), AlVIPR1 (AFG28559.1), PlPSP3 (AAA91584.1), TkSPRT (ABN04079.1), MaPR1B (AAC49831.1), MaPR1A (CAC95049.1), PcVCP1 (CAD20578.1), ThPRB1 (AAA34211.1), TvSP1 (AAO63588.1), TlSP1 (PTB78911.1), AmMLX (AAW21809.2), OoPII (CAA63841.1), and OoAOZ1 (AAM93666.1).

### 2.3. Analysis of TlSP1 Gene Expression

The *T. longibrachiatum* T6 mycelia parasitized on the surfaces of the cysts were collected at 12, 24, 48, 72, 96, 120, and 144 h after inoculation, and the mycelia grown on PDA medium without cysts was used as a control. For each sample, the total RNA was converted to cDNA using the RevertAid First Strand cDNA Synthesis Kit (Invitrogen), followed by quantitative real-time PCR (qRT-PCR) using the SYBR Green Premix Pro TaqHS qPCR Kit (Accurate Biotechnology (Hunan) Co., Ltd., Changsha, China). *Actin* (accession number: MN641014.1) was used as the internal reference gene for the determination of the target gene of *TlSP1* expression. The specific primers of *TlSP1* and the internal reference gene used for the qRT-PCR analysis are listed in Table 1. qRT-PCR was performed using the Thermo QuantStudio™ 5 thermal cycler (Thermo Fisher Scientific), and the relative gene expression was calculated using the 2^−ΔΔCT^ method [24]. Three biological replicates were performed for each amplification reaction.

### 2.4. Heterologous Expression of TlSP1 in P. pastoris X33

The *TlSP1* gene without the signal peptide was amplified by PCR using specific primers (*TlSP1-*F: TCTCGAGAAAAGAGAGGCTGAAGCTGCTCCAGCTGTTAACCACAAGTTGCCACAAGCTGTCCCAAACAAGTTTACATCG and *TlSP1-*R: CAAACTCAATGATGATGATGATGATGGTCGACAGCAGAGTTTCCGTTGAAGATGATGGTGTTTGGGGAACCGTTCA) and then fusion-integrated into the pPICZαA vector containing a 6× His tag. Next, pPICZαA-*TlSP1* was transduced into the expression strain *P. pastoris* X33 by electro-transformation, and *P. pastoris* X33 transformation experiments were performed as outlined by Wang et al. [25]. The presence of *TlSP1* in the *P. pastoris* X33 transformants were then confirmed by PCR using specific primers (*TlSP1*-F: 5′-ACTTCCCCAGCTAATGTTCC-3′ and 3′AOX: 5′-GCAAATGGTACATTCTGATACATCC-3′).

### 2.5. Induction, Expression, and Purification of Recombinant TlSP1 Protein

The inducible expression of the target proteins was carried out and optimized with reference to the instructions for the use of the pPICZαA expression system. Transformant colonies identified as positive during the procedure described in Section 2.4 were inoculated into buffered glycerol-complex medium (BMGY) and cultivated at 28.5 °C with agitation at 220 rpm until the OD_600_ reached the range of 4–6. Subsequently, the buffered methanol-complex medium (BMMY) was substituted for induction, and the fermentation mixture was subjected to oscillating cultivation at 28.5 °C and 220 rpm to trigger expression. The addition of 100% methanol occurred every 24 h to maintain a final concentration of 1%. Following expression induction, fermentation supernatants were collected at 24, 48, 72, 96, and 120 h time points for enzyme activity assessment. Upon determining the optimal induction time, 1 L of fermentation supernatant was collected, and the protein fermentation supernatant was concentrated by salting out using a saturated ammonium sulfate solution. Saturated ammonium sulfate was slowly added to reach a final concentration of 80% while stirring at 4 °C environment, and an ice bath was used overnight. Meanwhile, the sample was placed under centrifugation at 4 °C and 8000 rpm for 15 min, the supernatant was discarded, and the protein precipitate was solubilized with 1× phosphate-buffered solution (PBS) and purified enzymatically by Ni-NTA. The molecular weight of the recombinant protein was determined by SDS-PAGE [26].

### 2.6. Effects of Temperature on Activity and Stability of Recombinant TlSP1 Protein

The preparation of 1% casein (*w*/*v*) as a substrate was performed for the determination of the TlSP1 activity using the Folin reagent method [27]. The TlSP1 and substrate were subjected to different temperatures (40, 50, 60, 70, 80, and 90 °C) for 30 min to assess the impact of the temperature on enzyme activity [28]. All other conditions remained consistent with those employed for the enzyme activity determination under standard settings. The relative enzyme activity was calculated with the enzyme activity under optimal conditions set at 100%. Additionally, TlSP1 was pre-incubated at different temperatures (40, 50, 60, 70, 80, and 90 °C) for durations of 1, 3, 5, and 7 h. After rapid cooling to room temperature, the remaining enzyme activity was evaluated under standard conditions, with the relative enzyme activity determined using the enzyme’s activity under optimal conditions as the reference point (100%). 

### 2.7. Effects of pH on Activity and Stability of Recombinant TlSP1 Protein

To determine the optimal pH, the enzyme activity assays was conducted across a pH range of 3 to 10 at the optimal temperature. Different buffers were employed as follows: sodium acetate (pH 3.0–5.5), 0.1 M sodium phosphate (pH 6.0–7.5), 0.1 M Tris–HCl (pH 8.0–9.0), and 0.1 M glycine NaOH (pH 9.5–11.0). For the pH stability assessment, the enzyme solution was incubated at 4 °C for 12 h in various pH buffers, and the enzyme activity was measured at the optimal reaction temperature [28]. The relative enzyme activity was determined with the highest activity set at 100%.

### 2.8. Determination of Recombinant TlSP1 Protein Metal Ions

TlSP1 was pre-incubated with different metal ions, namely Zn^2+^, Ca^2+^, Mn^2+^, Mg^2+^, Hg^2+^, K^+^, Na^+^, Cu^2+^, Li^+^, and Fe^2+^, at a final concentration of 5 mM for 30 min and then the enzyme activity was determined [23]. The untreated enzyme was also used as a control to determine its activity as 100% and to analyze the effect of metal ions on the enzyme activity.

### 2.9. Determination of Various Inhibitors of Recombinant TlSP1 Protein

The impact of inhibitors, such as ethylenediaminetetraacetic acid (EDTA) and phenylmethylsulfonyl fluoride (PMSF), on enzyme activity was investigated. TlSP1 was subjected to incubation with final concentrations of 1 mM and 5 mM of these inhibitors. Subsequently, the residual enzyme hydrolysis activity was measured as a percentage of the enzyme hydrolysis activity observed in control samples without inhibitors [23].

### 2.10. Specificity of Various Substrates of Recombinant TlSP1 Protein

Reaction substrates including 1.0% (*w*/*v*) casein, gelatin, hemoglobin, collagen, BSA, and cysts were prepared and added to the enzyme solution. The enzymes’ degradation activity on various substrates was evaluated under optimal conditions. The relative activity of casein as a substrate was taken as 100% [29].

### 2.11. Determination of Kinetic Parameters

The kinetic parameter (K_m_ and V_max_) values for the purified TlSP1 were assessed using various casein substrate concentrations ranging from 0.8 to 10 mg·mL^−1^ at pH 8.0 and 50 °C. K_m_ and V_max_ were derived utilizing the Michaelis–Menten equation and the XY analysis function of the Origin 2021 software (OriginLab, Northampton, MA, USA).

### 2.12. Microscopic Observation of Effect of TlSP1 on Eggshells

Following the method described by Zhang et al. [10], cysts were surface-sterilized, crushed in a tissue grinder, and rinsed with sterile water through a 200-mesh sieve. Subsequently, they were transferred to a 500-mesh sieve, and the eggs were washed on the sieve. The concentration of the egg suspension was adjusted to 300–400 eggs·mL^−1^. Then, 10 μL of the egg suspension was extracted and mixed with 190 μL TlSP1. Additionally, 190 μL of sterile water and 1× PBS were added as controls. Each treatment and control were replicated six times. The experiment was conducted at 20 °C, and the changes in the eggshell were monitored and photographed using a Zeiss Axio Lab A1 microscope (Carl Zeiss, Suzhou, China) every 2 days [23].

### 2.13. Detection of TlSP1-Induced Callus Accumulation and Allergic Reaction in Wheat

Wheat seeds were surface sterilised with 1% sodium hypochlorite (NaOCl, 5 min) and sown in 10 cm diameter pots filled with sterile sandy soil. Seedlings were grown in an artificial climate chamber at 25 ± 0.5 °C with 16/8 h of light. When the wheat grew to 10–15 cm, the roots were rinsed with sterile water and treated with 30 mL of TlSP1 at a concentration of 1 μg·mL^−1^ for 24 h. Leaves were cut with scissors. The assessment of callus accumulation and allergic reactions in wheat induced by TlSP1 utilized a plant callus staining solution (Aniline blue method) and a plant tissue staining kit (Tepan blue method) from Beijing Solarbio Science & Technology Co., Ltd. (Beijing, China). Callus accumulation in wheat was observed using a fluorescence microscope, where green, fluorescent substances were detected at 200× magnification. Allergic reactions were identified through blue spots observed under a stereomicroscope at 40× magnification. The experimental setup used TlSP1 as treatment, sterile water and PBS serving as controls. Each experiment was replicated three times.

### 2.14. Study of TlSP1 Inducing Resistance to Cyst Nematode Disease in Wheat Using Root Splitting System

**Figure 1:**
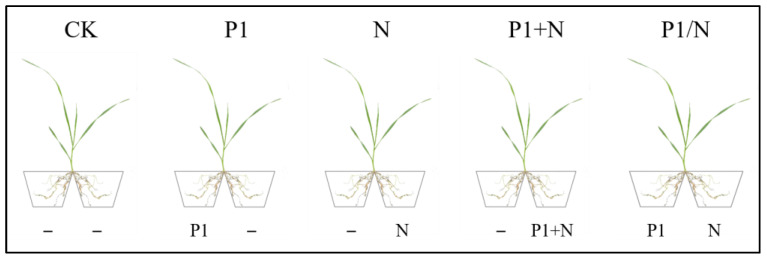
Schematic diagram of TlSP1-induced resistance to cyst nematodes in wheat studied in the split-root system.

The experiment included five treatments and was replicated three times. The wheat cultivar ‘Bailinong 207’, susceptible to *H. avenae*, was used in the experiment. Wheat seeds were surface-sterilized and sown in 10-cm-diameter pots containing sterile sandy soil. The seedlings were grown in an artificial climate chamber with an air temperature of 25 ± 0.5 °C and supplemental light/dark lighting of 16/8 h. The roots of 7-day-old wheat seedlings were cultivated and rinsed in sterilised water and divided into two parts, each planted in a 10-cm-diameter culture bowl containing sterilized sandy soil, with the root system buried (Figure 1). Various treatments were applied (CK: both sides were inoculated with 10 mL sterile water; P1: one side was inoculated with 10 mL TlSP1, while the other side was inoculated with 10 mL sterile water; N: one side was inoculated with 5 mL of egg suspension, while the other side was inoculated with 10 mL sterile water; P1+N: one side was inoculated with 10 mL TlSP1 followed by 5 mL egg suspension, while the other side was inoculated with 10 mL sterile water; P1/N: one side was inoculated with 10 mL TlSP1, while the other side was inoculated with 5 mL of egg suspension). After 21 days of treatment, the dry and fresh weights of both the shoot and root parts of the plants were measured. The roots of wheat treated with N, P1+N, and P1/N were stained with acid magenta to assess the nematode presence, and the number of nematodes in the wheat roots was determined.

### 2.15. TlSP1 Induces Defense-Related Gene Expression

At intervals of 7 days from the day 7, 14, and 21 after incubation, wheat subjected to the aforementioned root splitting system was sampled, snap-frozen in liquid nitrogen, and stored at −80 °C in an ultra-low-temperature refrigerator for future analysis. Root total RNA extraction was carried out using the Plant RNA Kit (OMEGA). Subsequently, qRT-PCR was conducted using the SYBR Green Premix Pro TaqHS qPCR Kit (Accurate Biotechnology (Hunan) Co., Ltd.). The expression changes of *TaPAL*, *TaCAT*, *TaSOD*, *TaPOD*, *TaC4H*, *Ta4CL2*, *TaCAD1*, *TaCAD12*, *TaNPR1*, *TaPR1*, *TaPR2*, *TaPR4*, *TaAOS2*, and *TaOPR3* genes were detected. The *TaEF-1α* gene was used as the internal reference gene. Table 2 shows the related genes and primer sequences. The relative expression of the relevant genes was analyzed using the 2^−ΔΔCt^ method [25].

### 2.16. Statistical Analysis

All numerical data above were expressed as the mean ± standard deviation (SD) of independent experiments. Statistical analysis was performed using the SPSS version 26.0 software (IBM Corp., Armonk, NY, USA). Statistical differences were compared using one-way analysis of variance (ANOVA) based on Duncan’s multiple range test. Differences were considered statistically significant when *p* < 0.05 and were plotted using the Origin version 2021 software.

## 3. Results

### 3.1. Isolation and Characterization of TlSP1 from T. longibrachiatum T6

**Figure 2:**
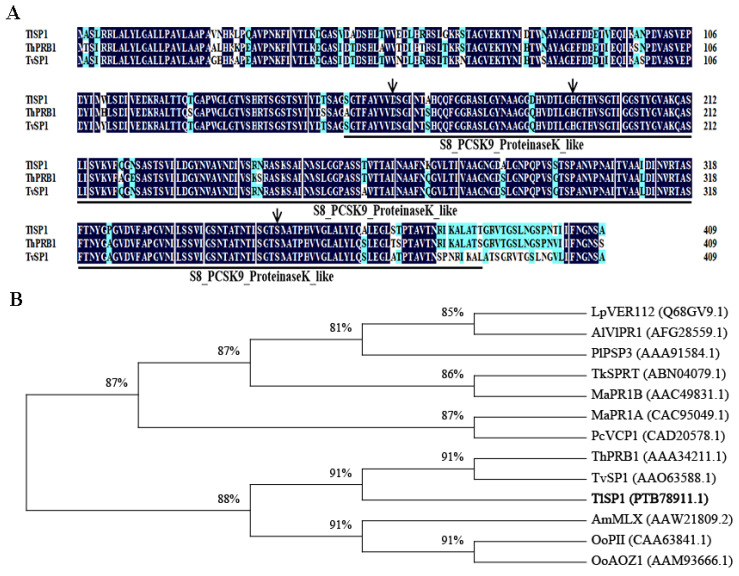
Multiple sequence alignment and phylogenetic analysis. (**A**) Multiple sequence alignment of TlSP1 with other species’ SPs. Thick solid lines and arrows represent highly conserved structural domains and the triad of Asp (D), His (H), and Ser (S) residues. The dark shaded areas indicate 100% sequence similarity, and the light blue shaded areas represent sequence similarity greater than 50%. (**B**) Phylogenetic tree of TlSP1 constructed using the neighbor-joining (NJ) method. Lp, *Lecanicillium psalliotae*. Al, *Akanthomyces lecanii*. Pl, *Purpureocillium lilacinum*. Ma, *Metarhizium anisopliae*. Pc, *Pochonia chlamydosporia*. Th, *T. harzianum*. Tv, *T. virens*. Tl, *T. longibrachiatum*. Am, *Arthrobotrys microscaphoides*. Oo, *Orbilia oligospora*. (Bootstrap = 1000).

The cyst-inducible *TlSP1* gene was cloned from *T. longibrachiatum* T6, which has a CDS with a full length of 1230 bp, encoding 409 amino acids, a molecular weight of 42.4 kDa, and an isoelectric point of 6.3. Bioinformatics analysis showed an instability index of 26.18, indicating a stable protein. The total mean value of hydrophilicity was 0.805, indicating a hydrophilic protein. In addition, there was a signal peptide at amino acids 1–20, and it was initially judged that TlSP1 might be a secreted protein. Secondary structure analysis showed that the protein consisted of 28.85% α-helices, 22.49% extended strands, 3.33% β-turns, and 41.32% random coils. Multiple sequence comparisons showed that the amino acid residues shared 91% similarity with both *T. virens* and *T. harzianum*. All three members contained the Asp (D) His (H) Ser (S) triplet and the conserved S8_PCSK9_ProteinaseK_like structural domain (Figure 2A). Further phylogenetic analyses showed that TlSP1 was closely related by co-clustering with TvSP1 and ThPRB1 in *T. virens* and *T. harzianum*, whereas other SPs clustered into other branches, which was consistent with the sequence comparison (Figure 2B).

### 3.2. Expression Patterns of TlSP1

**Figure 3:**
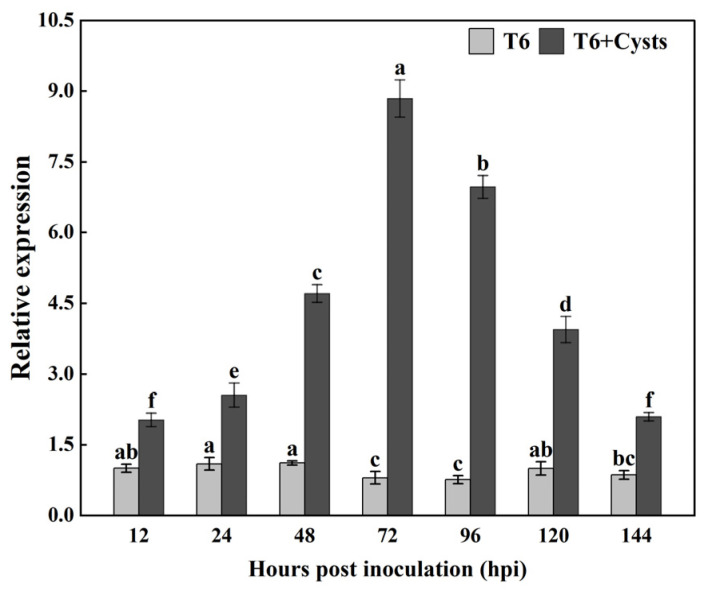
Analysis of the expression patterns of *TlSP1* gene. Data presented are means ± SD and columns labeled with different letters indicate significant differences at *p* < 0.05.

To detect the transcriptional profile of *TlSP1* in the interaction between T6 and cysts, the relative expression of the *TlSP1* gene in *T. longibrachiatum* T6 after inoculation with cysts was determined by qRT-PCR in this study. The results showed that the expression of the *TlSP1* gene was initially increased after inoculation with the cysts and peaked at 72 h, and it then gradually decreased with the increasing incubation time (Figure 3). After the interaction with the cysts, the *TlSP1* gene expression increased 2.02-, 2.55-, 4.71-, 8.85-, 6.97-, 3.94-, and 2.09-fold at 12, 24, 48, 72, 96, 120, and 144 h, respectively, compared with the control.

### 3.3. Heterologous Expression and Purification of TlSP1

**Figure 4:**
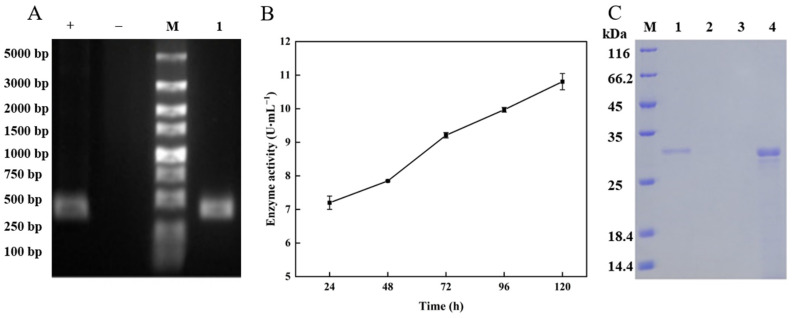
Identification of positive clone strains of *TlSP1* transformed into *P. pastoris* X33 and determination of protease activity at different times during fermentation of pPICZαA-*TlSP1*/X33 and purification of recombinant TlSP1 protein. (**A**) +: Positive control is pPICZαA-*TlSP1* recombinant vector; −: negative control is wild *P. pastoris* X33. 1: the PCR products of positive clones from *P. pastoris* X33 that have been transformed with the *TlSP1* gene. M: DL5000 DNA marker. (**B**) Protease activity at different fermentation times and (**C**) purification of recombinant TlSP1 protein, where M represents protein markers, 1 represents the recombinant protein crude enzyme solution sample, 2 represents the effluent sample, 3 represents 20 mM imidazole eluent, and 4 represents 500 mM imidazole eluent.

*TlSP1* recombinant *P. pastoris* X33 strains were detected by PCR using specific primers. As shown in Figure 4A, specific bands were detected in the recombinant colonies, whereas the presence of specific bands was not detected in the wild-type *P. pastoris* X33 strain, indicating that the *TlSP1* gene was successfully transformed into *P. pastoris* X33. The protease activity in the fermentation supernatant was measured after the recombinant strain was induced to ferment for different times (24, 48, 72, 96, and 120 h). The results showed that the protease activity increased significantly with the increasing fermentation time, reaching a maximum of 10.8 U·mL^−1^ at 120 h of induction (Figure 4B). The supernatant was purified using Ni-NTA after overnight dialysis, as shown in Figure 4C, and SDS-PAGE indicated that the purified enzyme solution formed a clear single band with an apparent molecular weight of approximately 32 kDa.

### 3.4. Characterization of TlSP1 Protein

**Figure 5:**
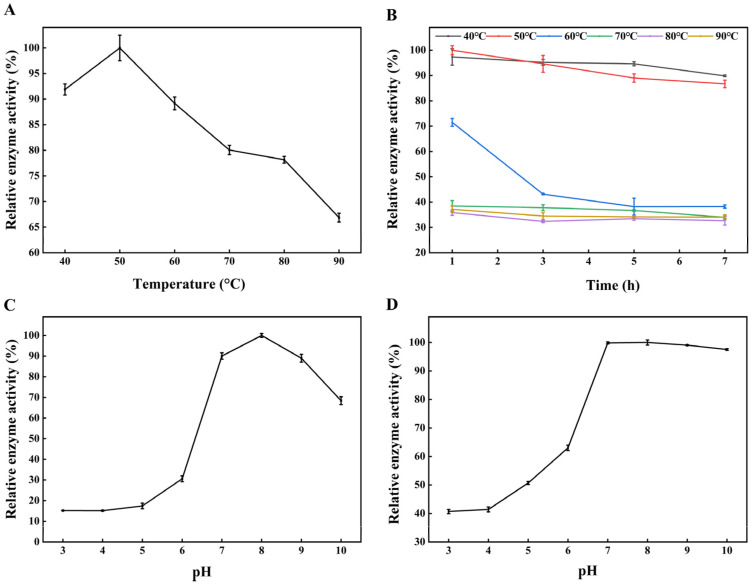
Effect of temperature and pH on enzyme activity of recombinant TlSP1 protein. (**A**) Optimal temperature, (**B**) thermal stability, (**C**) optimal pH, and (**D**) pH stability of TlSP1 protein.

The TlSP1 enzyme activity and stability were significantly affected by the temperature and pH. The enzyme activity peaked at 50 °C but declined at higher temperatures (Figure 5A). TlSP1’s thermal stability was maintained at over 80% activity after 7 h at 40 °C and 50 °C, with 30% activity remaining at temperatures above 50 °C (Figure 5B). The enzyme activity increased with the pH from 3 to 10, reaching a maximum at pH 8, and it was more stable under alkaline conditions, retaining over 90% activity at a pH above 7 (Figure 5C,D).

### 3.5. Effects of Metal Ions, Inhibitors, Substrate Specificity, and Kinetic Parameters of TlSP1

**Figure 6:**
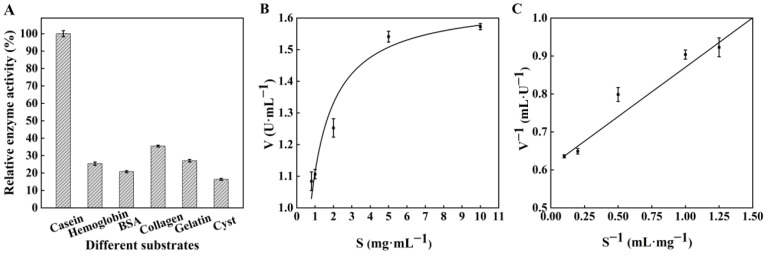
(**A**) Effect of substrate specificity and (**B**,**C**) kinetic parameters of TlSP1 enzyme activity. S: substrate concentration. V: enzyme-catalysed reaction rate.

The results showed that Cu^2+^, Mn^2+^, and K^+^ activated TlSP1, with Cu^2+^ being the most effective. The enzyme activity was determined using the inhibitors PMSF and EDTA and the results showed that PMSF inhibited TlSP1 more than EDTA at concentrations of 1 mM and 5 mM, respectively, indicating the presence of serine residues in the active site and classifying TlSP1 as a serine protease (Table 3). Figure 6A shows that casein is the preferred substrate for protease production. The V_max_ and K_m_ of the protease were 1.64 U·mL^−1^ and 0.43 mg·mL^−1^, respectively (Figure 6B,C).

### 3.6. Microscopic Observation of Effect of TlSP1 on Eggs

**Figure 7:**
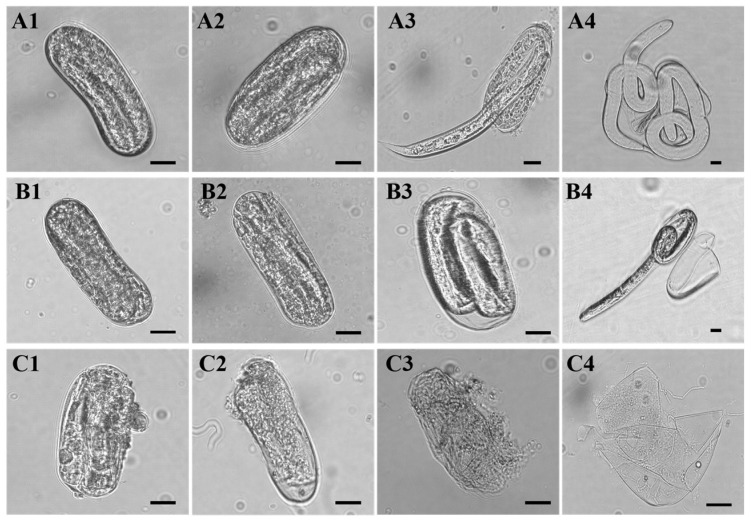
Morphological observation of eggs treated with recombinant TlSP1 protein. (**A1**–**A4**) represent eggs after treatment with sterile water at 4, 6, 8, and 10 days, respectively. (**B1**–**B4**) represent eggs after treatment with PBS at 4, 6, 8, and 10 days, respectively. (**C1**–**C4**) represent eggs after treatment with recombinant TlSP1 protein at 4, 6, 8, and 10 days, respectively. Bar: 20 μm.

Microscopic observations of recombinant TlSP1 acting on eggs showed that on the 4–6-day after treatment, the eggshells became thin and rough, leading to partial dissolution and rupture, and a small amount of the content was extravasated (Figure 7(C1,C2)). On the 8th day after treatment, the content extravasation was exacerbated (Figure 7(C3)). Most of the eggshells were dissolved on the 10th day after treatment (Figure 7(C4)). In contrast, the eggs in the PBS and sterile water groups showed no dissolution or content extravasation during the 4–8-day treatment period, and the J2s hatched on the 10th day were intact and active (Figure 7(A1–A4,B1–B4)).

### 3.7. Effect of TlSP1 on Callus Accumulation and Allergic Reaction in Wheat Seedlings

**Figure 8:**
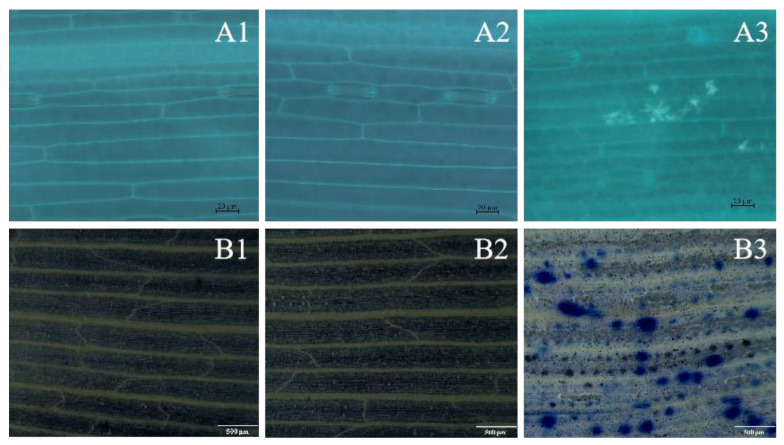
Effect of TlSP1 on callose accumulation and allergic reactions in wheat, depicted in photomicrographs. (**A1**–**A3**) illustrate callus accumulation, under 200× magnification: (**A1**) sterile water, (**A2**) PBS, (**A3**) TlSP1. Bar: 20 μm. (**B1**–**B3**) depict allergic reactions, under 40× magnification: (**B1**) sterile water, (**B2**) PBS, (**B3**) TlSP1. Bar: 500 μm.

Wheat seedlings treated with 1 μg·mL^−1^ TlSP1 and analyzed under a fluorescence microscope showed the accumulation of green fluorescent substances in the leaves (Figure 8(A3)), which was absent in the controls treated with sterile water and PBS (Figure 8(A1,A2)), suggesting that TlSP1 induces callus production. Additionally, blue spots indicating cell necrosis were observed near the leaf veins on TlSP1-treated leaves (Figure 8(B3)), whereas they were absent in the controls (Figure 8(B1,B2)), indicating that TlSP1 can induce allergic reactions in wheat.

### 3.8. Study of TlSP1-Induced Resistance to Cyst Nematodes in Wheat Using Split-Root System

#### 3.8.1. Effect of TlSP1 on Wheat Growth

**Figure 9:**
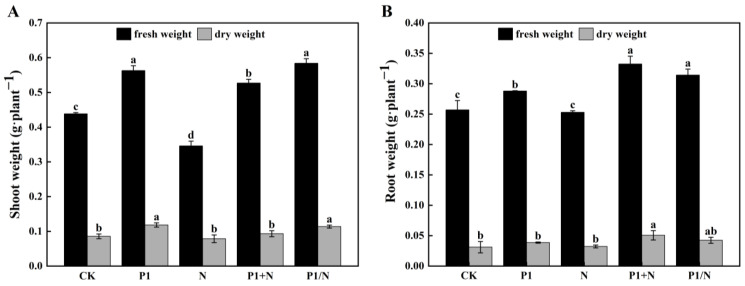
TlSP1 effect on wheat growth using the split-root method. (**A**) Shoot fresh and dry weights; (**B**) root fresh and dry weights. Different lowercase letters indicate significant differences at *p* < 0.05. Treatments are listed in Figure 1.

TlSP1 treatment increased the shoot fresh and dry weights by 28.38% and 38.08%, respectively, and the root fresh and dry weights by 12.07% and 24.12%, respectively, in the P1 treatment group compared to the CK group. In the combined TlSP1 and nematode treatment, the shoot fresh and dry weights increased by 20.19% and 8.88%, respectively, and the root fresh and dry weights increased by 29.39% and 62.70%, respectively, in P1+N compared to the control. Compared to the control, the TlSP1 treatment without nematodes (P1/N) increased the shoot fresh and dry weights by 33.17% and 32.83%, respectively, and the root fresh and dry weights by 22.30% and 36.33%, respectively. It is clear from these results that the treatment of wheat with TlSP1 has a positive effect on plant growth (Figure 9).

#### 3.8.2. Effect of TlSP1 on Nematode Invasion

**Figure 10:**
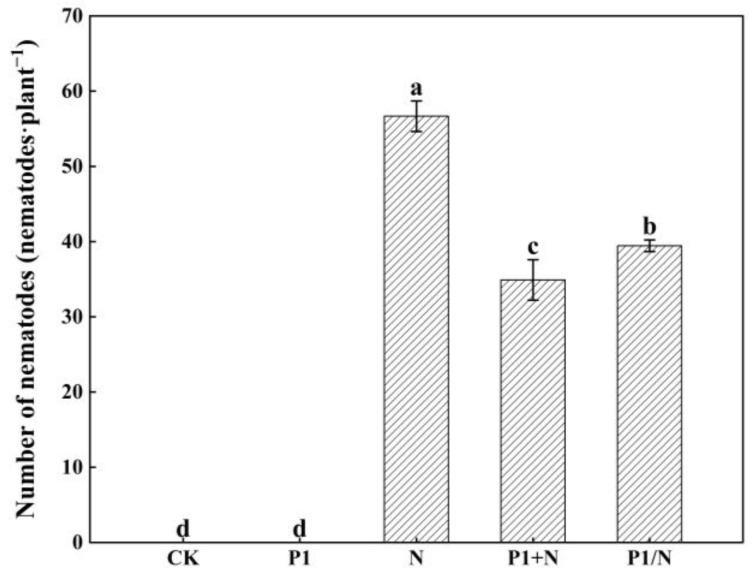
Effect of TlSP1 on nematode invasion. Data are presented as the means ± SD of three biological replicates. Different letters above the bars indicate significant differences (*p* < 0.05). Treatments are listed in Figure 1.

TlSP1 significantly induced disease resistance in the seedlings. Both the P1+N and P1/N treatment groups showed significantly fewer nematode invasions (*p* < 0.05) compared to the control (N). Compared to the control N group, the number of nematodes invading wheat roots was reduced by 38.43% in the P1+N, while the number of nematodes invading wheat roots was reduced by 30.4% in the P1/N (Figure 10).

#### 3.8.3. Expression Analysis of Defense-Related Enzyme Genes

**Figure 11:**
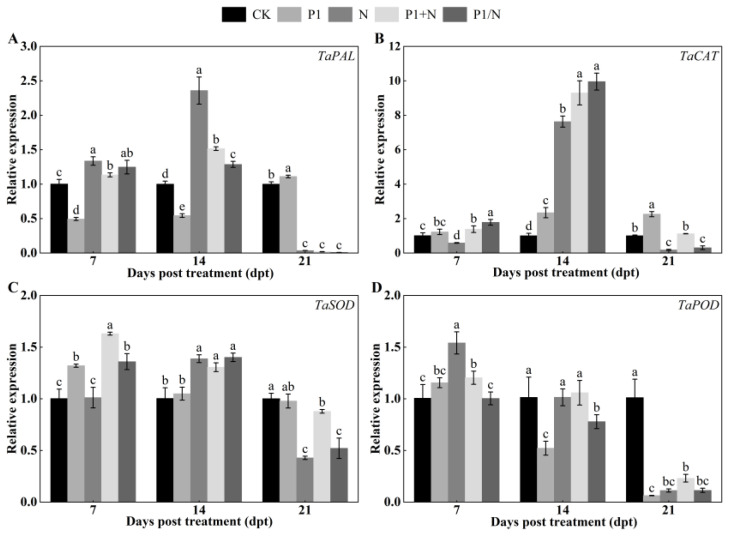
Effect of TlSP1 on (**A**) *TaPAL*, (**B**) *TaCAT*, (**C**) *TaSOD*, and (**D**) *TaPOD* relative expression at different days after treatment. Data are presented as the means ± SD of three biological replicates. Different letters above the bars indicate significant differences (*p* < 0.05). Treatments are listed in Figure 1.

The TlSP1 and nematode treatments significantly affected the defense-related enzyme genes expression in wheat seedlings roots (*p* < 0.05) (Figure 11). At 14 days, most genes were upregulated and peaked compared to the CK. The *TaPAL* gene expression was significantly upregulated and increased 2.36-, 1.51-, and 1.29-fold, respectively, in the N, P1+N, and P1/N groups compared to the CK at 14 days (Figure 11A). However, the expression of P1 in the treatment group decreased 0.54-fold. The *TaCAT* transcript levels were significantly upregulated and increased 2.34-, 7.63-, 9.30-, and 9.95-fold, respectively, in the P1, N, P1+N, and P1/N groups compared to the CK at 14 days (Figure 11B). The expression of the *TaSOD* gene was significantly upregulated and increased 1.05-, 1.39-, 1.31-, and 1.40-fold in the P1, N, P1+N, and P1/N treatment groups compared to the CK at 14 days, respectively (Figure 11C). The *TaPOD* gene expression in the P1 and P1/N treatment groups was downregulated and decreased 0.52- and 0.78-fold at 14 days compared to the CK. However, in the P1+N treatment group, the transcript level increased 1.06-fold, and the expression of N in the treatment group was not significantly different from that of the CK (Figure 11D).

#### 3.8.4. Expression Analysis of Lignin-Synthesis-Pathway-Related Genes

**Figure 12:**
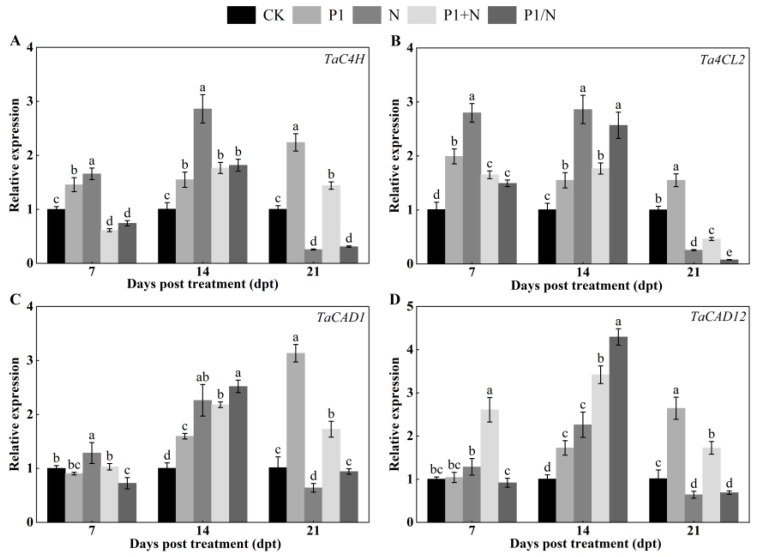
Effect of TlSP1 on (**A**) *TaC4H*, (**B**) *Ta4CL2*, (**C**) *TaCAD1*, and (**D**) *TaCAD12* relative expression at different days after treatment. Data are presented as the means ± SD of three biological replicates. Different letters above the bars indicate significant differences (*p* < 0.05). Treatments are listed in Figure 1.

The expression levels of TaC4H, Ta4CL2, TaCAD1 and TaCAD12 were significantly upregulated during the initial stage and continued to increase significantly at 14 days (Figure 12). Compared to the CK, the transcript levels of TaC4H in seedlings treated with P1, N, P1+N, and P1/N increased 1.55-, 2.86-, 1.77-, and 1.82-fold, respectively (Figure 12A). Similarly, the transcript levels of Ta4CL2 in the same treated seedlings increased 1.55-, 2.86-, 1.77-, and 2.57-fold, respectively (Figure 12B). Moreover, the transcript levels of TaCAD1 in the treated seedlings increased 1.60-, 2.26-, 2.18-, and 2.52-fold, while the transcript levels of TaCAD12 in the P1-, N-, P1+N- and P1/N-treated seedlings increased 1.72-, 2.26-, 3.42-, and 4.29-fold, respectively, compared to the CK (Figure 12C,D).

#### 3.8.5. Expression Analysis of SA Pathway-Related Genes

**Figure 13:**
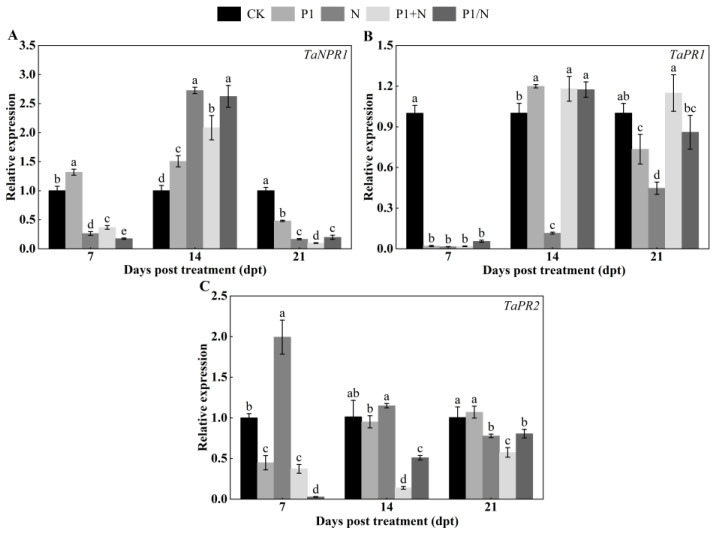
Effect of TlSP1 on (**A**) *TaNPR1*, (**B**) *TaPR1*, and (**C**) *TaPR2* genes of SA pathway. Data are presented as the means ± SD of three biological replicates. Different letters above the bars indicate significant differences (*p* < 0.05). Treatments are listed in Figure 1.

TaNPR1 plays a crucial role in the systemic acquired resistance (SAR) signaling pathway. After 14 days, the TaNPR1 expression was upregulated. The relative expression of TaNPR1 in the P1, N, P1+N, and P1/N treatment groups was significantly higher than in the CK treatment group and increased 1.51-, 2.73-, 2.08-, and 2.62-fold, respectively, and they were not infested with nematodes (Figure 13A). The TaPR1 gene expression showed a 0.12-fold decrease in the N treatment group at 14 days, while the P1, P1+N, and P1/N treatment groups increased 1.20-, 1.18-, and 1.17-fold, respectively (Figure 13B). For TaPR2 gene expression, the N treatment group showed a retreat at 14 days but it remained upregulated, with the P1, P1+N, and P1/N treatment groups showing 0.95-, 0.14-, and 0.51-fold decreases, respectively (Figure 13C).

#### 3.8.6. Expression Analysis of JA Pathway-Related Genes

**Figure 14:**
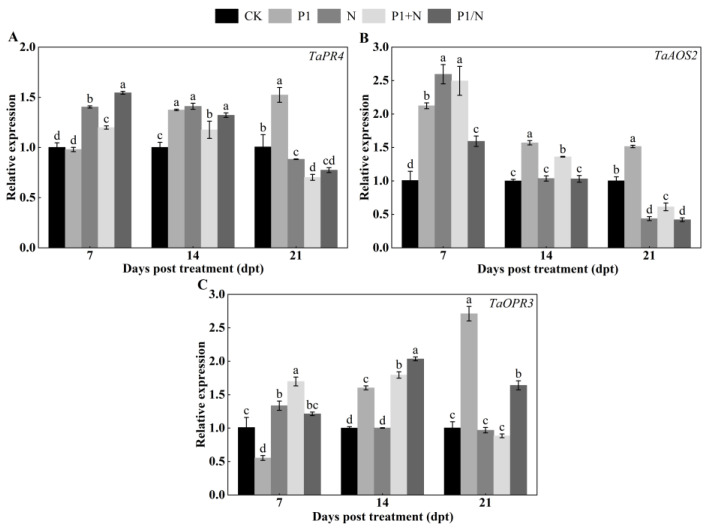
Effect of TlSP1 on (**A**) *TaPR4*, (**B**) *TaAOS2*, and (**C**) *TaOPR3* genes of JA pathway. Data are presented as the means ± SD of three biological replicates. Different letters above the bars indicate significant differences (*p* < 0.05). Treatments are listed in Figure 1.

The expression of *TaPR4* was upregulated after 14 days. The relative expression in the P1, N, P1+N, and P1/N treatment groups increased 1.37-, 1.41-, 1.18-, and 1.32-fold, respectively, and was significantly higher than that in the CK treatment groups, which were not infested with nematodes (Figure 14A). The expression of *TaAOS2* was upregulated after 14 days, with the relative expression in the P1, N, P1+N, and P1/N treatment groups being increased 1.57-, 1.04-, 1.36-, and 1.03-fold, respectively (Figure 14B). The expression of *TaOPR3* was upregulated after 14 days, with the relative expression in the P1, P1+N, and P1/N treatment groups being increased 1.60-, 1.79-, and 2.03-fold, respectively (Figure 14C).

## 4. Discussion

In our previous studies, *T. longibrachiatum* T6, a biocontrol fungus, exhibited inhibitory effects against a broad spectrum of plant pathogens, including fungi and nematodes [38,39,40]. This strain mitigates the damage inflicted by PPNs through various mechanisms, including direct parasitism, antibiosis, paralysis, and the secretion of lytic enzymes [10]. Many serine proteases with nematicidal activity have been purified from parasitic fungi, such as pSP-3 from *Paecilomyces lilacinus*, VCP1 from *Pochonia chlamydosporia*, and Ver112 from *Lecanicillium psalliotae*. They all have a similar molecular mass of approximately 32–33 kDa [41,42,43], which is very similar to that of TlSP1. Dunaevsky et al. [44] found that trypsin-like serine proteases secreted by *T. harzianum* remained active at 45–50 °C and pH 6.0–11.0. Aissaoui et al. [45] showed that the serine protease purified from *T. harzianum* maintained more than 62% of its initial activity between pH 5.0 and 10.0, with an optimum pH of 7.0, and showed residual activity of more than 85% at 30–50 °C. In this study, the optimum pH of TlSP1 was 8.0 and the optimum temperature range was 40–50 °C. The activity of TlSP1 remained at more than 80% after 7 h of incubation at the optimum temperature, indicating that the enzyme has strong thermal stability. Furthermore, TlSP1’s activity was significantly enhanced by various metal ions, particularly Cu^2+^, increasing the enzyme activity by 6.02%. PMSF exhibited stronger inhibitory effects than EDTA, indicating the presence of serine residues in the active site and classifying TlSP1 as a serine protease. TlSP1 displayed the ability to degrade a variety of substrates, including casein, hemoglobin, BSA, collagen, gelatin, and cysts. Consistent with our findings, extracellular hydrolytic enzymes, including serine protease, chitinase, lipase, and collagenase, are believed to play key roles in the infection process of parasitic fungi against PPNs [46]. All these proteases belong to the subtilisin-like serine protease family, with molecular masses typically ranging from 32 to 39 kDa. They exhibit similar characteristics, including the ability to target a diverse array of protein substrates, such as casein, gelatin, and eggshells. These enzymes play a crucial role in breaking down the nematode cuticle, leading to nematode mortality, and are considered significant virulence factors.

In this current study, treatment with TlSP1 significantly affected the nematode eggshells. Eggshells treated with TlSP1 became thin and rough, leading to partial dissolution and rupture, and a small amount of the content was extravasated. On the 8th day after treatment, the content extravasation was exacerbated, resulting in eggs that did not hatch normally. Most of the eggshells were dissolved on the 10th day after treatment. In contrast, the eggs in the control group showed no dissolution or leakage of their contents during the treatment period, with only a few J2s hatching in an intact and active state. TlSP1’s effects are attributed to the breakdown of the structural proteins of the nematode eggshells, which is essential for the survival and mobility of nematodes. Additionally, it may interfere with the nematodes’ digestive processes by degrading the proteins involved in nutrient absorption and digestion. This dual attack can effectively incapacitate and eventually kill the nematodes. Our findings are consistent with the study that reported that the purified trypsin-like serine protease PRA1 from *T. harzianum* inhibited the egg hatching of *M. incognita* [47]. Likewise, the subtilisin-like serine protease PRB1 from *T. atroviride* appeared to participate in virulence against *M. javanica*. Similar finding was observed in a study that reported that the crude extract of a solid-state ferment of *T. pseudokoningii* SMF2 displayed strong nematicidal abilities against *M. incognita* [18]. It could kill J2s and inhibit the hatching of early-stage eggs, but had no inhibitory effect on the hatching of last-stage eggs.

Previously, it was reported that metabolites from the fungus in liquid culture significantly reduce egg hatching and increase J2s’ mortality. Applying a culture suspension containing fungal spores more adversely affects J2 populations and enhances plant growth compared to fungal exudates [48]. Likewise, research on *Arabidopsis* shoot regeneration has revealed that the subtilase gene *AtSBT1.1* is expressed under conditions favorable for shoot regeneration [19]. Given that most subtilases are secreted proteins, serine protease *AtSBT1.1* might process extracellular growth factors or receptors involved in regeneration [49]. For instance, *AtSBT1.1* could act on peptide hormones like AtPSKs, which are known to enhance callus formation and undergo processing in their maturation [50]. Similarly, in this study, compared to the control groups, a 1.0 µg/mL concentration of TlSP1 protease controlled nematode infestation and increased the dry and fresh weights of the shoots and roots, respectively. In addition, TlSP1-treated seedlings showed the accumulation of green fluorescent substances in the leaves, which were absent in the controls treated with sterile water and PBS, suggesting that TlSP1 induces callus production. Moreover, blue spots indicating cell necrosis were observed near the leaf veins on TlSP1-treated leaves, which were absent in the controls treated with sterile water and PBS, indicating that TlSP1 can induce allergic reactions in wheat.

Serine protease has been explored for the control of PPNs by stimulating systemic defense mechanisms in plants, which accelerates the immune responses and reduces disease spread [51]. In this study, the serine protease TlSP1-induced resistance was confirmed via the split-root system. Both the P1+N and P1/N treatment groups showed significantly fewer nematode invasions compared to the control. TlSP1 degraded the nematode eggshells, causing high mortality. Both with and without the nematode treatments, the transcript levels of *TaPAL*, *TaSOD*, *TaPOD*, and *TaCAT* in TlSP1-treated seedlings were upregulated and significantly increased at the infection stage compared to the control. The overexpression of defense-related genes in TlSP1-treated seedlings enhanced the roots’ resistance to nematode invasion. Our findings are consistent with those who reported that the overexpression of serine protease gene *Tvsp1* in *T. virens* strains significantly increased the survival rate of cotton seedlings infected with *Rhizoctonia solani* by 15–32% over the wild-type strain [52]. In addition, the aspartic protease gene *papA* from *T. asperellum* T-203 was involved in mycoparasitism [53]. An egg-parasitic fungus, *P. chlamydosporia*, secreted several proteases in a submerged culture that hydrolyzed proteins from the outer layer of *M. incognita* eggshells, thereby exposing the chitin layer [22,54].

It was reported that during early *M. javanica* infection in tomato roots, SA signaling was downregulated, while JA-mediated responses were triggered by *Trichoderma* treatment, activating induced systemic resistance (ISR) [55]. In the current study, treatment with T6 serine protease TlSP1 and nematodes induced ISR, primarily via JA signaling pathways, and SAR through the SA and lignin synthesis pathways. Early in nematode infestation, genes linked to these pathways, including *TaC4H*, *Ta4CL2*, *TaCAD1*, and *TaCAD12* for lignin synthesis; *TaNPR1*, *TaPR1*, and *TaPR2* for the SA pathway; and *TaPR4*, *TaAOS2*, and *TaOPR3* for the JA pathway, were significantly upregulated. This upregulation was intensified at the infection stage. In the first phase, the presence of T6 serine protease TlSP1 stimulates faster SA-mediated defense responses, to protect the roots against nematode invasion. In the second phase, when *H. avenae* suppresses the JA-related defenses in the roots, T6 serine protease TlSP1 stimulates the expression of JA-dependent defenses, thus antagonizing the suppression of defenses mediated by *H. avenae*, which leads to a reduction in the development and reproduction of the nematodes. Hence, the defense induced by T6 serine protease TlSP1 can be mediated by the ISR or SAR pathways and also by a complex signaling network that connects SAR and ISR [51]. Our findings align with the results of earlier studies showing that SA and JA are signaling molecules influencing the protective system under biotic stresses, including that of phytoparasitic nematodes [56,57].

## 5. Conclusions

In this study, the *TlSP1* gene from *T. longibrachiatum* T6 was successfully cloned following induction by *H. avenae* cysts. The purified recombinant TlSP1 protein exhibited the ability to dissolve and degrade *H. avenae* eggshells. Additionally, it stimulated plant resistance, thereby enhancing the effective defense response against nematode invasion. These results enhance our understanding of the nematicidal genes and proteins in *T. longibrachiatum* T6, underscoring the potential of *TlSP1* as a biocontrol agent against nematodes.

## Figures and Tables

**Table 1:** Primers used to determine *TlSP1* gene relative expression by qRT-PCR analysis.

Primers Name	Primers Sequence (5′-3′)
*Actin*-F	TACATGGCTGGTCGTGATCTT
*Actin*-R	GCAGAGCTTCTCCTTGATGT
*TlSP1*-F	AGGCTCTTGAGGGTCTTAGT
*TlSP1*-R	GATAGTGTTAGGGCTCCTACATTC

**Table 2:** Primers used in qRT-PCR to induce defense-related gene expression.

Primer Name	Primer Sequence (5′-3′)	Reference
*TaEF-1α*-F	CAAGGGTGTGGAGAAGAAGG	[30]
*TaEF-1α*-R	AGCAGATACATAGATGGATTCAGG	
*TaPAL*-F	CGTCAAGAGCTGTGTGAAGATGG	
*TaPAL*-R	GGTAGTTGGAGCTGCAAGGGTC	
*TaCAT*-F	TGCCTGTGTTTTTTATCCGAGA	
*TaCAT*-R	CTGCTGATTAAGGTGTAGGTGTTGA	
*TaSOD*-F	CCGAGGTCTGGAACTACATCAC	
*TaSOD*-R	AGCCGAAATCCTTCTCGATCT	
*TaPOD*-F	TTGTGGTGGCGGTGGTAGTGG	[31]
*TaPOD*-R	CGAAGCAGTCGTGGAAGTGGAG	
*TaC4H*-F	GAAGAAGCTGGTGAGCACCA	[32]
*TaC4H*-R	TGATGTTCTCGATGATGTAG	
*Ta4CL2*-F	CAAGGAGCTGCAGGATACATCA	
*Ta4CL2*-R	GTCGACGATCTTGAGCTCCG	
*TaCAD1*-F	AAAAGCAGAGGGAGAAAGAGC	[33]
*TaCAD1*-R	AACGGAAACAGGTCACAAATACAT	
*TaCAD12*-F	AACGTGACCAAGTTCAAGGC	
*TaCAD12*-R	TTCAGCCCGTGGTACTTTACAT	
*TaNPR1*-F	GCTCACAGAAGGGCAGACAA	[34]
*TaNPR1*-R	GCTTAGCGGCGATGTGAAGA	
*TaPR1*-F	CTGGAGCACGAAGCTGCAG	[35]
*TaPR1*-R	CGAGTGCTGGAGCTTGCAGT	
*TaPR2*-F	CTCGATACATCGGTAACGACCAG	
*TaPR2*-R	GCGGCGATGTACTTGATGTTC	
*TaPR4*-F	ACACCGTCTTCACCAAGATCGACA	[36]
*TaPR4*-R	AGTACATGGATCAGTCTCAGTGCTCA	
*TaAOS2*-F	CAACTTCAACACGCTCAACGA	[37]
*TaAOS2*-R	GGAGCTGGAATATGAGCCACTT	
*TaOPR3*-F	AAGATATTGTTGCCTGATGGTTCA	
*TaOPR3*-R	CAGCTTGGCGATATTGCTCAA	

**Table 3:** Effects of metal ions and chemical reagents on the activity of the TlSP1 protein.

Reagent	Concentration (mM)	Relative Activity (%)
CK ^a^	-	100.00 ± 2.77 ^b^
Mg^2+^	5	94.13 ± 1.77 ^c^
Fe^2+^	5	91.85 ± 2.41 ^cd^
Ca^2+^	5	89.37 ± 4.04 ^cde^
Cu^2+^	5	106.02 ± 5.19 ^a^
Mn^2+^	5	101.30 ± 2.44 ^b^
Zn^2+^	5	88.27 ± 3.51 ^de^
Na^+^	5	93.26 ± 1.61 ^c^
K^+^	5	101.83 ± 2.21 ^ab^
Hg^+^	5	82.55 ± 1.67 ^f^
Li^+^	5	86.44 ± 0.59 ^ef^
EDTA	1	91.96 ± 3.08 ^cd^
	5	92.11 ± 1.49 ^cd^
PMSF	1	61.60 ± 1.20 ^g^
	5	24.04 ± 0.83 ^h^

^a^ The activity of the TlSP1 protein without any addition was considered as CK. Data are the mean ± SD. Columns followed by different letters were significantly different at *p* < 0.05 based on the Duncan’s multiple range test.

## Data Availability

The original contributions presented in the study are included in the article, further inquiries can be directed to the corresponding authors/first author.
